# IS*1111 *insertion sequences of *Coxiella burnetii*: characterization and use for repetitive element PCR-based differentiation of *Coxiella burnetii *isolates

**DOI:** 10.1186/1471-2180-7-91

**Published:** 2007-10-18

**Authors:** Amy M Denison, Herbert A Thompson, Robert F Massung

**Affiliations:** 1 Coordinating Center for Infectious Diseases, Division of Viral and Rickettsial Diseases, Rickettsial Zoonoses Branch, Centers for Disease Control and Prevention, Atlanta, GA, USA

## Abstract

**Background:**

*Coxiella burnetii *contains the IS*1111 *transposase which is present 20 times in the Nine Mile phase I (9Mi/I) genome. A single PCR primer that binds to each IS element, and primers specific to a region ~500-bp upstream of each of the 20 IS*1111 *elements were designed. The amplified products were characterized and used to develop a repetitive element PCR genotyping method.

**Results:**

Isolates Nine Mile phase II, Nine Mile RSA 514, Nine Mile Baca, Scottish, Ohio, Australian QD, Henzerling phase I, Henzerling phase II, M44, KAV, PAV, Q238, Q195 and WAV were tested by PCR and compared to 9Mi/I. Sequencing was used to determine the exact differences in isolates which lacked specific IS elements or produced PCR products of differing size. From this data, an algorithm was created utilizing four primer pairs that allows for differentiation of unknown isolates into five genomic groups. Additional isolates (Priscilla Q177, Idaho Q, Qiyi, Poker Cat, Q229 and Q172) and nine veterinary samples were characterized using the algorithm which resulted in their placement into three distinct genomic groups.

**Conclusion:**

Through this study significant differences, including missing elements and sequence alterations within and near IS element coding regions, were found between the isolates tested. Further, a method for differentiation of *C. burnetii *isolates into one of five genomic groups was created. This algorithm may ultimately help to determine the relatedness between known and unknown isolates of *C. burnetii*.

## Background

*Coxiella burnetii *is a gram-negative, obligate, intracellular bacterium that causes Q fever in humans. Infection occurs through inhalation of contaminated aerosols and fewer then ten organisms is sufficient to seed an infection [1]. Q fever can manifest as an acute infection characterized by a flu-like febrile illness, headache, and atypical pneumonia and the recommended treatment is doxycycline. A chronic infection is typified by endocarditis and/or hepatitis with poor prognosis [2].

Initial studies examining restriction fragment length polymorphisms (RFLP) in chromosomal and plasmid DNA from seven *C. burnetii *isolates revealed genetic heterogeneity among the isolates [3]. In subsequent research, RFLP patterns analyzed via sodium dodecyl sulfate-polyacrylamide [4] or pulsed field gel electrophoresis [5] allowed for the creation of six distinct genomic groups (I through VI). Isolates from acute disease patients could be placed in genomic groups I, II and III, while groups IV and V contained isolates from chronic disease patients. Furthermore, isolates placed in genomic groups I, II and III were shown to contain the plasmid QpH1, group IV isolates to contain the plasmid QpRS, and group V isolates are plasmidless [5], though QpRS-homologous sequences are located in chromosomal genome sequences of the group V isolates [6,7]. The group VI isolates were initially reported to contain the plasmid QpDG, though newer experiments suggest these may actually possess QpH1, and QpDG containing isolates were shown to be avirulent in guinea pigs [8,9]. Additional studies have characterized a variety of isolates using RFLP/pulsed field gel electrophoresis with differing restriction enzymes [10], multispacer sequence typing [11], multiple loci variable number of tandem repeats analysis [12,13], and microarrays [14] which have discriminated isolates into as many as 36 distinct genotypes.

With the completion of the genome sequence of Nine Mile Phase I (9Mi/I), 20 unique copies of the IS*1111 *transposase were identified [15]. Previous work describing the insertion sequence noted that the coding region of the transposase is bounded by two sets of terminal inverted repeats at each end, which were previously referred to as the inner and outer inverted repeats [16]. The inner inverted repeats are thought to represent the termini of the IS element. It is predicted that IS*1111 *forms a circular intermediate, whereby the inner inverted repeats are brought closer together to form a strong promoter which increases transposase expression. The outer inverted repeats are believed to be chromosomal sequences which form a stem-loop structure that acts as a recognition site for insertion of the IS element but are not part of the IS element itself. This chromosomal target sequence is present more than 50 times throughout the 9Mi/I genome [17]. Sequence differences in the various copies of IS*1111*, most of which are present in the loop of the stem-loop recognition site, led to its further subdivision into IS*1111*A, IS*1111*B and IS*1111*C [16]. We sought to compare the locations of IS*1111 *in a variety of isolates of *C. burnetii *to that of the 20 copies present in 9Mi/I using a PCR-based method. Because of the high copy number of the IS element in the *C. burnetii *genome and the unique regions adjacent to each element, this has proved to be a useful tool in differentiating isolates of *C. burnetii *into groups, much like the six genomic groups proposed previously [4,5]. We present an algorithm which distinguishes members from five of the six genomic groups using four primer pairs and a two-step PCR cycling program.

## Results

PCR analysis included 14 isolates, 10 of which had previously been characterized as members of genetic groupings I through V [Nine Mile phase II (9Mi/II), Nine Mile RSA 514 (RSA 514), Ohio, Australian QD, Q195, Henzerling RSA 343 (Henz I), Henzerling, RSA 331 (Henz II), M44, KAV, PAV, Q238 and WAV], and 4 [Nine Mile Baca (9Mi/Baca), Scottish, Q238 and WAV] which had not been genotyped [4]. Our repository lacks the group VI Dugway isolates, thus these were not included. The analysis was completed for each isolate for each of the 20 IS elements present in 9Mi/I using the consensus primer IS1111-1 paired with a unique primer upstream of each of the IS elements. The presence or absence of certain IS elements was generally consistent among members of a particular genogroup, but allowed their differentiation from members of other genogroups. The results from each of these 14 isolates are provided (see Additional file 1) along with the element's orientation and nucleotide position in 9Mi/I [GenBank: AE016828], and GenBank accession numbers for the sequences determined in this study.

### Genomic group I isolates

9Mi/II, RSA 514, Ohio, and Australian QD appeared to have all 20 IS elements and their products were indistinguishable from each other and 9Mi/I by this method. The isolates 9Mi/Baca and Scottish, which have not been genotyped previously, also had all 20 IS elements and were placed within this group.

### Genomic group II isolates

**Figure 1:**
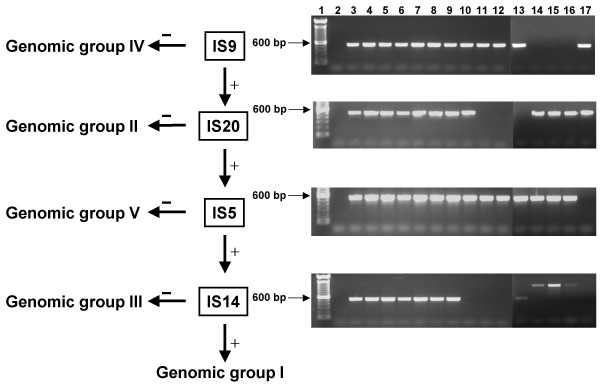
Proposed algorithm flowchart and agarose gel photographs of corresponding PCR products. To discriminate isolates of *C. burnetii *using the algorithm shown, PCR is performed using the primer pairs depicted in boxes and negative PCR reactions allow for the discrimination of isolates into the genomic groups shown. Agarose gels of PCR products using primer pairs (from top to bottom) IS 9 and IS1111-1, IS 20 and IS1111-1, IS 5 and IS1111-1, and IS 14 and IS1111-1. Lanes 1, 100 bp ladder; 2, negative control; 3, 9Mi/I; 4, 9Mi/II; 5, RSA 514; 6, 9Mi/Baca; 7, Scottish; 8, Ohio; 9, Australian QD; 10, Q195; 11, Henz I; 12, Henz II; 13, M44; 14, KAV Q154; 15, PAV Q173; 16, Q238; 17, WAV.

PCR products amplified using DNA from isolates Henz I, Henz II, and M44 were identical in size to 9Mi/I for 15 of the 20 IS elements (2, 3, 5, 6, 7, 8, 9, 11, 12, 13, 15, 16, 17, 18, and 19). IS elements 14 and 20 were confirmed absent in these isolates by sequencing, though our M44 isolate also possessed a subpopulation which contained IS 14 (Fig 1). In regards to IS 14, the deletion totaled 1,374 bp, which corresponds exactly to the predicted length of the IS element [17]. Also within this region, Henz I and Henz II, but not M44, contain a point mutation. The IS 20 deletion in these three isolates totaled 2,246 bp.

Amplicons to IS elements 1 and 4 were present but of larger size than 9Mi/I. In all three group II isolates, indels consisted of small deletions and slightly larger insertions. The IS 10 region, however, was quite unique in that PCR products using primer pairs IS 10 and IS1111-1 produced a product of incorrect size. Upon amplification with primer pairs 1269R and 1273R, which correspond to genes flanking the IS 10 element in 9Mi/I, products were obtained. Sequence data indicated that the site where the IS 10 primer anneals is missing due to a deletion of 1,650 bp in these isolates, but that the IS element is indeed present.

### Genomic group III isolate

A single isolate from genomic group III, Q195, was available in our repositories for analysis. There were no noticeable differences in amplicon sizes between 9Mi/I and Q195 for 15 of the 20 IS elements (2, 3, 5, 6, 7, 8, 9, 10, 12, 13, 15, 16, 17, 18, and 19). The only IS element which Q195 lacked was IS 14. Sequencing of this region identified a genomic deletion of 1,374 bp.

Amplicons of larger size were produced to IS elements 1, 4 and 20. Upon sequencing of these PCR products, each was missing 3 nucleotides and an identical sequence of 26 nucleotides was inserted in its place in all cases. The PCR product for IS 11 was smaller for Q195 in comparison to 9Mi/I and was found to be lacking 8 bp.

### Genomic group IV isolates

Two isolates from chronic Q fever patients exhibiting endocarditis, KAV and PAV, have previously been placed into group IV [4]. Q238 is believed to be an isolate derived from the same patient as PAV, though it has not been genotyped previously. These three isolates were examined by PCR using the primer pairs to each of the 20 IS elements described above. No amplicon size differences were seen for 10 of the IS elements (2, 5, 6, 8, 11, 12, 13, 17, 19 and 20) when compared to 9Mi/I.

Analysis of IS 7 and IS 9 in the KAV, PAV and Q238 isolates showed that both of these IS elements were missing. Sequencing revealed the absence of 1,458 bp and 1,341 bp, respectively, compared to the IS 7 and IS 9 elements in the 9Mi/I genome. Similarly, IS elements 14, 15, and 16 were missing in each of the genomic group IV isolates because of identical deletions of 1,374 bp.

No products of the correct size were obtained from isolates KAV, PAV and Q238 with primers for IS 10. A long PCR product obtained using primers 1269R and 1273R was sequenced and an IS element was found in this region, although the annealing site for the IS 10 primer was eliminated. The sequence of DNA to which the IS element is connected corresponds to 9Mi/I nucleotide positions 1222860–1222964. The sequence is then interrupted by 133 bp of sequence that does not match sequence in 9Mi/I, but does match sequence found in the Dugway 7E9-12 strain [GenBank: AAQI00000000].

Products of IS 18-specific PCR in each of the three genomic group IV isolates appeared to be slightly larger than that of 9Mi/I. Upon sequencing of the KAV product, however, this appeared to be a consequence of primer mismatch to another IS element in the genome. The sequence data obtained [GenBank: DQ882594] corresponded to an IS element and a novel sequence not present in the 9Mi/I genome. However, this sequence was found in the Dugway 7E9-12 strain. Using long PCR to flanking genes and DNA from the three isolates, no products were obtained, though PCR of each of the flanking genes (data not shown) indicated they are present somewhere in the genome, suggesting that in these isolates, this region of the genome has undergone rearrangement compared to 9Mi/I.

PCR to IS elements 1, 3, and 4 produced products from KAV, PAV and Q238 differing in size relative to 9Mi/I. These variations were due to numerous indels ranging from 1 bp to 32 bp. The indels in both IS 1 and IS 3 resulted in frame shifts in the predicted IS1111 encoded protein.

### Genomic group V isolate

WAV had not been characterized previously but was hypothesized to be a member of genomic group V, as it could be distinguished from each of the other isolates at a variety of IS elements. It was indistinguishable from 9Mi/I for 12 of the 20 IS elements (2, 3, 6, 8, 9, 10, 11, 12, 13, 16, 17, and 18). Six nucleotides were missing in WAV in the products amplified from IS elements 1, 4, and 20 when compared to 9Mi/I and an identical 21-bp sequence was present in its place in each case.

No amplicon was generated using primers to IS elements 5, 7, 14, 15 and 19. Sequencing confirmed the absence of IS 5, 14 and 15 due to deletions of 1,374 bp, and of IS 7 due to a 1,458 bp deletion. Lastly, PCR to IS 19 using DNA from WAV did not result in an amplicon. Long PCR using primers 1960R and 1693R resulted in a product larger than that from 9Mi/I (data not shown). Sequencing elucidated that a rearrangement had occurred in this area of the WAV genome, but was not pursued further as it was evident that an IS element was no longer present in this portion of the WAV genome.

### Creation and testing of an algorithm to differentiate *C. burnetii *strains

Using the information gained above, an algorithm was derived which discriminated each of the five genomic groups of isolates tested (Fig. 1). Because only group IV heart valve isolates (KAV, PAV and Q238) lacked products to IS 9, Henz I, Henz II, and M44 (group II) lacked IS 20, and WAV (group V) lacked IS 5, PCR to these isolates could be used to discriminate these groups. Isolates positive to each of these IS elements could then be further tested using PCR to IS 14, which separates Q195 (group III) isolates from group I isolates. PCR to IS 14 also serves to confirm that group II, IV and V isolates, which also lack this IS element, are grouped appropriately.

Further, each of the primer pairs used in the algorithm was tested using a two-step PCR program, and all products were successfully amplified (Fig. 1). The algorithm and the two-step PCR was then tested with additional isolates of *C. burnetii *and veterinary samples previously shown to be PCR positive for the agent. This included two isolates previously characterized, Priscilla Q177 (group IV) and Q229 (group V), four isolates of unknown genomic group (Idaho Q, Qiyi, Poker Cat and Q172), and nine veterinary samples from a single outbreak in 2005 on a goat ranch in Colorado. As expected, Priscilla Q177 and Q229 could be differentiated appropriately due to the lack of PCR products when using primers to IS 9 and IS 5, respectively. Q172 could also be placed into group V based on its lack of product to IS 5. PCR to IS 14 was also negative in these isolates, confirming that Priscilla Q177, Q229, and Q172 lacked this IS element as predicted. The remaining isolates (Idaho Q, Qiyi and Poker Cat) produced PCR products with all primer pairs and therefore were identical to 9Mi/I in our assay and placed within group I (data not shown). Six of the veterinary samples lacked PCR products to IS 9 and IS 14, placing them in genomic group IV. Three of the veterinary samples, one from placenta and two from feces, did not give PCR products using the above conditions. Since these three samples were positive for *C. burnetii *when tested with a real time-PCR assay that amplifies the IS*1111 *element, it was suspected that an inhibitor was present. By using a master mix that has demonstrated increased sensitivity in our hands (PuReTaq Ready-To-Go PCR Beads, GE Healthcare, Fairfield, CT) in lieu of Qiagen *Taq *Master Mix, PCR products were successfully obtained such that these samples were also shown to be members of genomic group IV.

## Discussion

In this study, PCR amplification and DNA sequencing of the IS*1111 *transposase elements in the genome of *C. burnetii *and their surrounding sequence has been used to extensively characterize these elements in multiple isolates, and as target regions for inter- and intra-genomic analysis. In some isolates, IS element PCR resulted in products either larger or smaller than expected based on the 9Mi/I genome and these were generally due to small indels in the isolates tested. Numerous IS elements present in the 9Mi/I genome were absent in the isolates evaluated, mostly due to large deletions. Of the 25 deletions which could be completely characterized by sequencing, 16 were 1,374 in length. Larger deletions were detected at IS 20 (2,246 bp) in the group II isolates and at IS 7 (1,458 bp) in the group IV and V isolates, while a smaller deletion (1,341 bp) was detected at IS 9 in the group IV isolates. The 1,374-bp length corresponds exactly with the predicted length of the IS element [17], demonstrating that the IS element has likely failed to integrate into these sites thus far. The presence of larger deletions may indicate that the IS element inserted into certain sites and subsequently excised, taking additional genomic sequence with it or causing genomic rearrangements when doing so. Numerous nucleotide polymorphisms were also seen in the stem-loop portion of the IS insertion target sites (data not shown) which may render the sites unrecognizable by the transposase, or alternatively, the IS element simply may not have inserted into these IS recognition sites. The sequence data demonstrate that, where polymorphisms were noted, the stem of the stem-loop structure is most times preserved while changes are evident in the loop region. An understanding of target site recognition and insertion mechanisms will help to determine the importance of the nucleotides within the loop region. If it is found that the overall structure but not specific nucleotide sequence is key to recognition, the division of IS*1111 *into IS*1111*A, IS*1111*B and IS*1111*C, as previously suggested based on sequence variation, may be unnecessary [16].

Analysis of the M44 isolate was complicated by evidence of a mixed population when analyzing IS 14. This result could indicate that the isolate is truly a mixed population, which is quite possible since it was never cloned. Previous work from our laboratory supports this hypothesis, as prior analysis of this isolate by an indirect immunofluorescence assay demonstrated epitopes reactive with both phase I- and phase II-specific antibodies [18]. However, this result could also occur if IS*1111 *is actively transposing in the genome, in which case circular intermediates should be detectable. PCR to determine if circular forms of IS*1111 *were present in 9Mi/I did not result in a product, though the linear form of IS*1111 *appeared to be expressed as judged by reverse transcriptase-PCR analysis (data not shown). It may be that expression of this linear form leads to an actively transposing IS element at low levels that we could not detect by PCR. The IS element appears rather stable, however, in that all 9Mi/I derived isolates have IS elements identically placed with no copies having been eliminated, though additional copies could be present and would not be detected by the methods used in this study. Certainly further research into the mechanism and frequency of IS*1111 *transposition is warranted.

The inter-genomic comparisons based on our IS element analysis showed very good correlation with RFLP analysis that was previously used to divide *C. burnetii *isolates into six distinct genomic groups [4]. Therefore, this analysis was used to develop a PCR-based genotyping assay which identifies an isolate as within one of the genomic groups, I through V. The assay was effective in discriminating genomic groupings in veterinary samples as well as purified isolates of *C. burnetii*. While most veterinary samples could be differentiated using the conditions initially set forth, an inhibitor was found to block amplification in three of the veterinary samples, as PCR products to all IS elements, even those conserved in all isolates tested, were not produced. Inhibition was easily circumvented by using a different master mix. It should be noted that inhibition occurred with both of the fecal samples that were tested. DNA from fecal samples is typically more difficult to amplify due to the presence of inhibitors and other factors, and if DNA from feces will be used for genotyping, extractions should be performed using protocols designed especially for feces [19]. Therefore, it is recommended that a control utilizing primers to a conserved IS element or a housekeeping gene also be included in the run to check for inhibition of the reaction. The DNA from any group I isolate should be used as a positive size control in applications of the algorithm, as group I isolates produce a product with all four algorithm primer pairs. Further, negative PCR results to any of the IS elements in the algorithm should also be verified by sequencing the region to ensure that mutations or deletions in primer binding regions are not at fault and that the IS element is truly lacking from this region. Sequencing of PCR products elucidated single nucleotide polymorphisms in IS element regions (data not shown) which could distinguish KAV from PAV, though no differences were observed between Henz I and Henz II or PAV and Q238. Sequencing was also effective at determining which IS elements were being amplified, and in some cases, sequences not found in the 9Mi/I genome were identified next to IS elements. It is interesting to note that in the group IV isolates, IS 10 and IS 18 primers annealed to sequences present in the Dugway 7E9-12 genome but not in Nine Mile. The origin of the Dugway strain and how the group IV isolates came to share genomic sequence with this strain, evolutionarily speaking, is intriguing. While other genomic changes undetectable by this method are surely present in these isolates, which would allow for further discrimination of isolates into a larger number of genotypes, the ability to quickly identify members of genomic groups I through V is useful. Perhaps a combination of rapid PCR-based methods such as the algorithm presented here, and VNTR (variable number tandem repeats) analysis [12,13] or MLS (multispacer sequence) typing [11] for potentially higher resolution, will prove optimal.

It is interesting to note that the Qiyi isolate, typed as genomic group I in this study, was reportedly isolated from the bone marrow of a patient with chronic Q fever [20]. This represents a relatively novel finding, and supports a previous report that identified an isolate characterized as within genomic group I from a chronically infected human in France [10]. In contrast, most isolates from chronically infected humans have been classified into genomic groups IV and V. Clearly, further examination of the genomic composition and evolutionary relatedness of isolates from both acute and chronic human infections is warranted.

## Conclusion

This study has begun to shed new light on the variations of IS elements within the genome of multiple isolates and the mechanism of IS element insertion in the *C. burnetii *genome. It also presents an algorithm which may provide a useful tool for grouping unknown isolates. Further, with the additional use of sequencing in these regions, a multitude of information can be learned about new isolates which may be used to determine their relatedness to known isolates of *C. burnetii*.

## Methods

### Bacterial isolates and veterinary samples

The isolates studied, their source, and passage history are shown in Table 1. Isolates 9Mi/I, 9Mi/II, RSA 514, 9Mi/Baca, Scottish, Ohio, Australian QD, Q195, Henz I, Henz II, M44, KAV Q154, PAV Q173, Q238 and WAV, which represent members of genomic groups I through V, were initially used to create the algorithm presented below. Isolates Priscilla Q177, Idaho Q, Qiyi, Poker Cat, Q229 and Q172 were employed to test the algorithm, as were nine veterinary samples from a single outbreak at a goat ranch in Colorado in 2005. Of the nine samples, five were taken from placental tissue, two from feces, and two from milk.

**Table 1:** Original source and passage history of *C. burnetii *isolates used in this study.

Genomic group					
				
RFLP^*a*^	IS*1111 *PCR^*b*^	Plasmid type	Isolate (Phase)^*c*^	Original source	Passage history^*d*^
I	I	QpH1	9Mi/I/C7 (I)	Montana, tick, 1935	307GP/1TC/1EP
I	I	QpH1	9Mi/II/C4 (II)	Montana, tick, 1935	304GP/90EP/1TC/4EP
I	I	QpH1	9Mi/RSA 514 (II)	Montana, tick, 1935	307GP/1TC/1EP/343 days GP/5EP
I	I	QpH1	Ohio (I)	Ohio, cow's milk, 1956	5EP/2GP/2EP
I	I	QpH1	Australian QD (II)	Australia, human blood, ~1939	177EP
ND^*e*^	I	QpH1	Scottish (ND)	Unknown	Unknown
ND	I	QpH1	Idaho Q (ND)	Unknown	Unknown
ND	I	ND	9Mi/Baca (II)	Montana, tick, 1935	307GP/1TC/1EP/4091 days TC
II	II	QpH1	Henzerling RSA 343 (I)	Italy, human blood, 1945	6GP/25EP/1GP/4EP
II	II	QpH1	Henzerling RSA 331 (II)	Italy, human blood, 1945	6GP/25EP/1GP/36EP
II	II	QpH1	M44 (II)	Greece, human blood, ~1945	1GP/86EP/20GP/50MP/5EP
III	III	QpH1	Q195 (ND)	Idaho, goat	Unknown
IV	IV	QpRS	KAV Q154 (I)	Oregon, human heart valve, 1976	1GP/3EP
IV	IV	QpRS	PAV Q173 (I)	California, human heart valve, 1979	2EP
IV	IV	QpRS	Priscilla Q177 (I)	Montana, goat cotyledon, 1980	Unknown
ND	IV	QpRS	Q238 (ND)	Human heart valve, 1979	3EP
V	V	Plasmidless	Q229 (I)	Human heart valve, 1982	Unknown
ND	V	ND	WAV (ND)	Human heart valve	Unknown
ND	V	ND	Q172 (ND)	Human placenta	3MP/2EP
ND	I	ND	Qiyi (ND)	China, human bone marrow, 1962	Unknown
ND	I	ND	Poker Cat (ND)	Nova Scotia, 1987	Unknown

^*a *^As determined in previous studies [4, 5].

^*b *^As determined in this study.

^*c *^Phase of isolate as determined by complement block titration, M.G. Peacock, Rocky Mountain Laboratories, Hamilton, Montana [4].

^*d *^Numbers indicate passage in: GP, guinea pig; TC, tissue culture; EP, embryonated eggs; MP, mice

^*e *^Not determined

### DNA isolation, PCR and sequencing analysis

DNA was extracted from purified bacteria or crude yolk sac preparations using the QIAamp DNA Mini Kit (Qiagen, Valencia, CA). DNA was extracted from veterinary samples using the QIAamp DNA Blood Kit (Qiagen), following the manufacturers protocol for extraction from tissue samples. A PCR primer (IS1111-1) was designed to anneal to a sequence conserved in all IS elements within the 9Mi/I genome, and was paired with each of 20 primers selected to bind to unique sequences approximately 500 bp upstream from each of the 20 IS elements. The sequence of each primer used in this study is listed in Table 2, along with the predicted amplicon size. For initial screening as well as algorithm testing, PCR reactions were performed in a 25 μl volume using the Qiagen *Taq *PCR Master Mix kit, 0.2 μM of each primer, and 0.5 μl of template DNA. For initial screenings, PCR cycling conditions consisted of an initial denaturation at 95°C for 3 min, 35 cycles each consisting of 30 s at temperatures of 95°C, 55°C, and 72°C, and a final extension for 7 min at 72°C. Reactions testing the algorithm (IS elements 5, 9, 14 and 20) utilized 2 μl of template DNA and were subjected to cycling conditions of an initial denaturation at 95°C for 3 min, 40 cycles each consisting of 30 s at temperatures of 95°C and 68°C, and a final extension for 7 min at 72°C. PCR products were visualized using ethidium bromide-stained 1.2% agarose gels.

**Table 2:** Primers, their target, sequence, primer pairings and predicted amplicon size of primer pair.

Target	Primer name	Sequence (5' to 3')	Paired primer set and predicted amplicon size
Conserved for each IS element	IS1111-1	ACTGCGTTGGGATACCCATC	NA^*a*^
IS1	IS1	TAATGGGCGACCAAGTCGA	IS1111-1; 501 bp
IS2	IS2	GGCTGAATGAATGCCTTCCA	IS1111-1; 501 bp
IS3	IS3	AAAAAGACCCTTCAGGTAATGGAA	IS1111-1; 477 bp
IS4	IS4	TTCCGCCATGACCAACTTC	IS1111-1; 519 bp
IS5	IS5	GTCGGTCAACGTCGTCACAT	IS1111-1; 515 bp
	556F	AACCGTGGCTGAGCGATC	IS5; NA
IS6	IS6	CCATTTGCAATAGCCGTGAA	IS1111-1; 477 bp
IS7	IS7	GCGGTTTTGGTACAATACTGTCATT	IS1111-1; 571 bp
	1091F	TGCAGGACATATTGTTGATGTCAC	IS7; NA
IS8	IS8	GGCAAGGATGACGAATGGAT	IS1111-1; 471 bp
IS9	IS9	GCCTCAGCCGATTTCGAG	IS1111-1; 492 bp
	1219F	CCCGAAGGTATCCATAACTCTGG	IS9; NA
IS10	IS10	GTGCGGCATTCGTGGTAGA	IS1111-1; 481 bp
	1269R	GATTTCCATCGACGTCCGC	1273R; NA
	1273R	TTCAGTTTATAGCGGCAGGACC	IS1111-1; NA
IS11	IS11	AAAGCCGTTCCCTACCGTG	IS1111-1; 481 bp
IS12	IS12	ACGTAGTTTGGAAAGGATACCCG	IS1111-1; 482 bp
IS13	IS13	GATGAGCGTCCACCACTGAA	IS1111-1; 537 bp
IS14	IS14	TGCTACCAACAGACTTACGGCA	IS1111-1; 481 bp
	1701R	TAAACACGAGGCGCTAGGTCA	IS14; NA
IS15	IS15	AACGCCCATGTGAGAAACATC	IS1111-1; 531 bp
	1718F	ACGGGTGTTCAAGCAGCC	IS15; NA
IS16	IS16	ATGACTTCTGATAGGGACTGTGCTC	IS1111-1; 511 bp
	1761F	GCCGTTGATCTTGCCGATAA	IS16; NA
IS17	IS17	CGAATCGCATTGGCACAGT	IS1111-1; 477 bp
IS18	IS18	CGTTTGCTGCTATACGACATGAA	IS1111-1; 532 bp
IS19	IS19	GTCGCCCAGGAGTGTTTCTG	IS1111-1; 531 bp
	1960R	ACAAAGACGACTCAGTTCGGC	1963R; NA
	1963R	CGATGTCTGCCTTTAGGAGGTC	1960R; NA
	1963R-seq	GTAGAAAGAGTCTCTCTTG	NA
IS20	IS20	ACGTCAATTACATCGAGCATTCA	IS1111-1; 470 bp
	1987F	AGGGCCATCTTGACCTGGA	1993F; NA
	1993F	CGCACTGAGCGCATTGAC	1987F; NA

^*a *^Not applicable

For isolates which lacked specific IS elements, the upstream primer was paired with additional PCR primers (see Table 2) to adjacent downstream genes based on the 9Mi/I genome sequence [GenBank: AE016828]. In some instances, new upstream primers were also needed to determine the extent of the deleted DNA (IS10, IS18 and IS19), sometimes resulting in large PCR amplicons. When PCR amplicons were predicted to be larger than 1.5 kb, long PCR was performed. These reactions were carried out in 25 μl volumes using Extensor High-Fidelity PCR Master Mix (ABgene Inc. USA, Rochester, NY), 0.2 μM of each primer, and 0.5 μl of template DNA. PCR cycling conditions consisted of an initial denaturation at 94°C for 2 min, 10 cycles each consisting of 94°C for 10 sec, 55°C for 30 sec, and 68°C for 8 min, followed by 20 cycles each consisting of 94°C for 10 sec, 55°C for 30 sec, and 68°C for 8 min +10 sec/cycle, with a final extension for 7 min at 68°C. Products were visualized using ethidium bromide-stained 0.8% agarose gels.

In instances where amplicons were of a different size than those present in 9Mi/I or where products were obtained using primers to genes adjacent to a missing IS element, DNA sequencing was performed. PCR products obtained above were processed through a DNA Clean & Concentrator-5 column (Zymo Research Corp., Orange, CA) and eluted in 16 μl of water. Sequencing reactions were performed with 1 μl of purified PCR product, one of the primers used to generate that PCR product, and the BigDye Terminator v3.1 Cycle Sequencing kit (Applied Biosystems, Foster City, CA). Unincorporated dye terminators were removed with a DyeEx 2.0 Spin Kit (Qiagen), the eluate was dried in a vacuum centrifuge, and the pellet suspended in 10 ul of Hi-Di formamide (Applied Biosystems). Sequencing reactions were analyzed in an ABI Prism 3100 Genetic Analyzer, and the Genetics Computer Group Wisconsin Package Version 10.3 (Accelrys Inc., San Diego, CA) was used for subsequent analysis of DNA sequences.

## Authors' contributions

AMD carried out all experimental elements of the study and drafted the manuscript. HAT and RFM conceived the study, participated in its design and helped to draft the manuscript. All authors have read and approved the final manuscript.

## Supplementary Material

Additional file 1 Presence (+) or absence (-) of IS elements in various isolates of *C. burnetii*; Table includes a description of each of the 20 IS elements and notes whether the element was present or absent in each of the isolates tested, as well as Genbank accession numbers for deletions and mutations discovered during this study. Click here for file
